# Evaluation of a rapid immunoenzymatic screening kit (SAMElife™) for drugs of abuse detection in hair

**DOI:** 10.1093/jat/bkag053

**Published:** 2026-06-18

**Authors:** Valentina Martini, Stefania Ortu, Cristiana Stramesi, Claudia Vignali, Luca Morini

**Affiliations:** Department of Public Health, Experimental and Forensic Medicine, University of Pavia, Pavia, PV, 27100, Italy; Department of Public Health, Experimental and Forensic Medicine, University of Pavia, Pavia, PV, 27100, Italy; Department of Public Health, Experimental and Forensic Medicine, University of Pavia, Pavia, PV, 27100, Italy; Department of Public Health, Experimental and Forensic Medicine, University of Pavia, Pavia, PV, 27100, Italy; Department of Public Health, Experimental and Forensic Medicine, University of Pavia, Pavia, PV, 27100, Italy

## Abstract

The performance of the SAMElife™ rapid hair screening kit was evaluated for the detection of drugs of abuse in 31 anonymized hair samples, encompassing 217 analyses for morphine, cocaine, amphetamines, buprenorphine, oxycodone, fentanyl, and methadone. Screening results were compared with confirmatory gas chromatography–mass spectrometry (GC–MS) and liquid chromatography–tandem mass spectrometry (LC–MS/MS) analyses. The kit demonstrated high specificity (100%) and good sensitivity (81.8%) for basic drugs, with four false negatives observed for cocaine, while no false positives were recorded. Detection of Δ9-tetrahydrocannabinol (THC) was unreliable, yielding uniformly positive results regardless of confirmatory analysis. The automated workflow, requiring only hair cutting and no prior decontamination, provides a rapid and practical approach for preliminary screening in routine toxicology laboratories. Despite limitations in THC detection and the small number of positive samples for some analytes, the SAMElife™ kit offers a reliable tool to reduce the number of samples requiring confirmatory analysis. Further evaluation on larger populations and optimization of the THC assay are recommended.

## Introduction

Hair analysis is widely employed in forensic and clinical toxicology, since drugs and their metabolites become incorporated into the hair shaft during growth, allowing retrospective assessment of drug exposure over extended time periods ranging from weeks to several months [1–4]. Given the large number of samples processed in routine toxicological laboratories, the exclusive use of confirmatory chromatographic methods would be time-consuming and analytically demanding. Therefore, immunochemical screening assays are commonly used as a first analytical step to identify presumptive positive samples and reduce the number of specimens requiring confirmatory analysis. However, immunoassays applied to hair matrices present inherent challenges in sensitivity and specificity. These challenges are mainly related to the low concentrations of drugs typically incorporated into hair, the complexity of analyte extraction from the keratin matrix, and the possibility of matrix-related interferences or antibody cross-reactivity, all of which may compromise assay sensitivity and specificity [5, 6]. In particular, low analyte concentrations may result in false-negative findings, whereas external contamination and limited antibody selectivity may contribute to false-positive results [5]. Furthermore, many immunochemical methods originally developed for conventional biological matrices (e.g. urine or blood) require methodological adaptation before application to hair analysis [7, 8].

The SAMElife™ system was considered worthy of evaluation because, unlike traditional immunoassays requiring extensive sample preparation or prolonged extraction procedures, it provides a rapid and largely automated workflow specifically designed for hair testing, requiring only minimal sample handling before analysis [7, 9, 10]. If sufficiently reliable, such an approach could potentially reduce laboratory turnaround time and decrease the number of samples requiring confirmatory chromatographic analyses in routine toxicological settings [9, 10].

Moreover, positive results lack medico-legal validity without confirmatory analysis using mass spectrometry–based techniques, such as gas chromatography–mass spectrometry (GC–MS) or liquid chromatography–tandem mass spectrometry (LC–MS/MS), for definitive identification [6]. The aim of the present study was to evaluate the performance of a rapid immunoenzymatic screening kit (SAMElife™) for the detection of selected drugs of abuse in hair samples and compare the screening results with confirmatory GC–MS and LC–MS/MS analyses.

## Materials and methods

### Chemicals

All solvents were of high-performance liquid ­chromatography (HPLC) grade and obtained from Carlo Erba Reagents SRL (Milan, Italy). Certified standards and deuterated internal standards were purchased from Cerilliant Corporation (Merk, Milan, Italy). MSTFA (N-methyl-N-(trimethylsilyl)trifluoroacetamide), HFPOH (1,1,1,3,3,3-hexafluoro-2-propanol), and PFPA (pentafluoropropionic acid) were obtained from Sigma-Aldrich (Milan, Italy). Water was purified using a Milli-Q filtration system (Merck Millipore, Milan, Italy) according to the manufacturer’s specifications.

### Hair samples

Thirty-one anonymized hair samples were purposively selected from specimens submitted for routine toxicological analysis in order to include confirmed positive cases for the target analytes evaluated by the SAMElife™ kit. Selection was based on previous confirmatory GC–MS and/or LC–MS/MS results to ensure representation of different drugs of abuse. In addition, six samples from subjects not expected to be positive for drugs of abuse and confirmed negative by chromatographic analyses were included as negative controls. A subset of 15 samples from the study population used for the basic drugs panel was additionally evaluated for Δ9-tetrahydrocannabinol (THC). Hair strands were cut as close as possible to the scalp and stored at room temperature until analysis.

### Sample preparation

For immunoenzymatic screening with SAMElife™, hair strands were cut into ∼1 cm segments and processed directly in the kit’s automated system, avoiding a washing procedure.

For GC–MS and LC–MS/MS analyses, hair samples were decontaminated by washing the samples with 1 mL of methanol, followed by vortex mixing and centrifugation for 5 min. Samples were then dried under a nitrogen stream until complete solvent evaporation and manually cut into approximately 1–2 mm fragments before analysis.

### Screening analysis

Screening analysis was performed using the commercially ­available SAMElife™ rapid hair testing kit according to the manufacturer’s instructions. The kit is designed to detect morphine, cocaine, amphetamines, buprenorphine, oxycodone, fentanyl, methadone, and THC. The screening results were interpreted according to the manufacturer’s recommended cut-off values: 0.2 ng/mg for morphine/opiates, 0.5 ng/mg for cocaine and amphetamines, 0.04 ng/mg for buprenorphine and fentanyl, 0.2 ng/mg for methadone and oxycodone, and 0.1 ng/mg for THC. The analytical protocol was the following**:** The T-HAIR processor homogenizes the hair segments with the extraction solution under controlled conditions. The homogenate is transferred onto the test cartridge, where chromophore-conjugated monoclonal antibodies specific for each analyte bind their targets. A colorimetric signal appears as reactive bands, which is read within 5–15 min depending on the analyte. The automated system standardizes extraction and homogenization, and no prior solvent washing is required.

### Confirmatory analysis

All samples were analyzed using both GC–MS and LC–MS/MS as appropriate for each target analyte, following validated laboratory protocols. Confirmatory analyses were ­quantitative and based on routinely applied laboratory procedures for hair toxicology. Briefly, GC–MS analyses were performed on an Agilent 6890 Series GC coupled with an HP 5973 mass spectrometer, using a 12 m × 0.2 mm fused silica capillary column with 5% phenyl methylpolysiloxane stationary phase. GC–MS was used for the detection of morphine, THC, amphetamines, and cocaine. LC–MS/MS analyses were conducted on an Agilent 1290 Series LC coupled with a 4000 Q-TRAP mass spectrometer, using a Kinetex C18 column (100 × 2.1 mm, 2.6 μm). LC–MS/MS was used for the detection of fentanyl, methadone, buprenorphine, and oxycodone. Chromatographic and mass spectrometric conditions, including mobile phases and acquisition parameters, were optimized for each analyte according to established laboratory procedures, and the method sensitivity satisfied the thresholds suggested by the recommendations of the Society of Hair Testing (SoHT) [11]. Detailed analytical conditions are routinely applied in our laboratory for quantitative hair toxicology analysis. Positive confirmatory results were interpreted according to the cut-off thresholds recommended by the SoHT and routinely adopted by our laboratory: 0.2 ng/mg for opiates, methadone, and amphetamines, 0.5 ng/mg for cocaine (with benzoylecgonine ≥0.05 ng/mg and BE ≥5% of cocaine concentration), 0.05 ng/mg for cannabinoids, and 0.005 ng/mg for buprenorphine[11]. For analytes not specifically covered by SoHT recommendations (e.g. fentanyl and oxycodone), interpretation was based on validated laboratory criteria.

## Results

The SAMElife™ rapid hair screening kit was evaluated on 31 anonymized hair samples, totaling 217 analyses across seven target drugs of abuse: morphine, cocaine, amphetamines, buprenorphine, oxycodone, fentanyl, and methadone. The study population included 25 purposively selected samples from routine toxicological analysis and 6 samples from subjects not expected to be positive for drugs of abuse, which were confirmed negative by GC–MS/LC–MS/MS and included as controls.

The performance of the screening kit for individual basic drugs is summarized in Table 1.

**Table 1:** Performance of SAMElife™ rapid hair screening kit compared to confirmatory GC–MS/LC–MS/MS analyses.

Analyte	True positives (VP)	True negatives (VN)	False positives (FP)	False negatives (FN)	Notes
Morphine	2	29	0	0	–
Methadone	4	27	0	0	–
Buprenorphine	1	30	0	0	–
Fentanyl	2	29	0	0	–
Oxycodone	1	30	0	0	–
Cocaine	8	19	0	4	4 FN = doubtful cases
Amphetamines	–	31	0	0	Not evaluated (no positive samples)

Diagnostic performance was evaluated by calculating sensitivity, specificity, overall accuracy, positive predictive value (PPV), and negative predictive value (NPV) using standard equations based on true positives (TP), true negatives (TN), false positives (FP), and false negatives (FN). (Sensitivity = TP/(TP+FN); Specificity = TN/(TN+FP); PPV = TP/(TP+FP); NPV = TN/(TN+FN); Accuracy = (TP+TN)/(TP+TN+FP+FN)).

Considering all 217 screening analyses collectively, the kit demonstrated:

- Sensitivity: 81.8% (18/22 TP detected)
- Specificity: 100% (195/195 TN correctly identified)
- Overall accuracy: 98.2%
- PPV: 100%
- NPV: 97.99%

The four FN occurred in cocaine-positive samples and were ­classified as “doubtful” by three independent operators when not following the manufacturer’s interpretation guidelines. No FP were observed for any analyte.

For THC, a subset of 15 samples from the study population was tested, including samples expected to be both positive and negative for cannabinoids based on prior confirmatory toxicological findings. All yielded positive results on the screening kit, whereas confirmatory GC–MS analysis identified only 4 positive samples and 11 negative samples. Because the screening assay classified all samples as positive, meaningful estimation of specificity and negative predictive performance was not possible. These findings indicate poor discriminatory performance of the kit for THC detection under the present experimental conditions.

## Discussion

The SAMElife™ kit exhibited high specificity and good sensitivity for the detection of major basic drugs of abuse in hair. Its rapid workflow, requiring only sample cutting without prior decontamination, represents a practical advantage for routine toxicological screening, particularly when processing large numbers of specimens. Compared with traditional immunoassay methods adapted for hair matrices, such as competitive enzyme-linked immunosorbent assay or fluorescence polarization immunoassay, rapid screening platforms like SAMElife™ provide a more streamlined workflow with minimal sample preparation, which can be especially beneficial in high-throughput laboratory settings [12, 13]. Previous evaluations of hair immunoassays have reported variable sensitivity, particularly for analytes such as cocaine and amphetamines, due to matrix interferences and cross-reactivity [7, 14]. In line with these findings, the present study observed perfect specificity across all basic drugs but reduced sensitivity for cocaine, reflecting the broader trend reported in hair immunoassay studies. While basic drugs were reliably detected, the kit showed poor discriminatory performance for THC, with all screened samples testing positive despite only a minority being confirmed by GC–MS. This finding is consistent with known limitations of hair immunoassays for cannabinoids. Reliable cannabinoid detection in hair remains challenging, as many commercially available immunoassays demonstrate inconsistent performance for THC, likely due to its low incorporation into hair and generally low analyte concentrations [15, 16]. Although a prior washing/decontamination step is not required by the SAMElife™ protocol, in the present study hair samples were nevertheless washed as a precautionary measure to minimize possible external contamination. Therefore, the uniformly positive THC screening results are unlikely to be solely explained by superficial contamination and may instead reflect limitations in the assay’s discriminatory performance for cannabinoids. These findings underscore the necessity of confirmatory mass spectrometry for cannabinoid analysis and highlight the need for further optimization of the THC assay. Future studies will be specifically aimed at further investigating and minimizing potential sources of interference or contamination affecting THC screening performance. In agreement with the manufacturer and based on the present findings, efforts are currently underway to optimize the SAMElife™ assay for cannabinoids in order to improve analytical performance and reduce the occurrence of false-positive results. Additional validation studies will therefore be conducted to assess the performance of the optimized THC assay under routine toxicological conditions.

Limitations include the relatively small number of TP samples for most analytes, except cocaine. The kit’s performance for THC was limited, yielding uniformly positive results regardless of actual sample status.

Overall, the device can serve as a reliable preliminary screening tool to reduce the number of samples requiring confirmatory GC–MS or LC–MS/MS analysis. Nevertheless, confirmatory chromatographic techniques remain essential for definitive identification. Further evaluation on a larger sample population is recommended, and the THC assay requires optimization before routine implementation.

## Conclusions

The SAMElife™ rapid hair screening kit demonstrated high specificity and good sensitivity for major basic drugs of abuse, providing a rapid and practical tool for preliminary screening in routine toxicology laboratories. Its workflow, requiring minimal sample preparation, allows efficient processing of large sample volumes while reducing the number of specimens needing confirmatory GC–MS or LC–MS/MS analysis. Limitations include inadequate performance for THC detection and the relatively small number of positive samples evaluated. Further studies on larger populations are needed, and optimization of the THC assay is recommended prior to routine implementation.

## Conflicts of interest

None declared.
